# Regional Neuroplastic Brain Changes in Patients with Chronic Inflammatory and Non-Inflammatory Visceral Pain

**DOI:** 10.1371/journal.pone.0084564

**Published:** 2014-01-08

**Authors:** Jui-Yang Hong, Jennifer S. Labus, Zhiguo Jiang, Cody Ashe-Mcnalley, Ivo Dinov, Arpana Gupta, Yonggang Shi, Jean Stains, Nuwanthi Heendeniya, Suzanne R. Smith, Kirsten Tillisch, Emeran A. Mayer

**Affiliations:** 1 Gail and Gerald Oppenheimer Family Center for Neurobiology of Stress, University of California Los Angeles, Los Angeles, California, United States of America; 2 Pain and Interoception Imaging Network (PAIN), University of California Los Angeles, Los Angeles, California, United States of America; 3 Department of Medicine, University of California Los Angeles, Los Angeles, California, United States of America; 4 Department of Psychiatry, University of California Los Angeles, Los Angeles, California, United States of America; 5 Brain Research Institute, University of California Los Angeles, Los Angeles, California, United States of America; 6 Division of Digestive Diseases, University of California Los Angeles, Los Angeles, California, United States of America; 7 Ahmanson Lovelace Brain Mapping Center, David Geffen School of Medicine, University of California, Los Angeles, California; 8 Department of Bioengineering, University of California Los Angeles, Los Angeles, California, United States of America; 9 University of Michigan School of Nursing, Ann Arbor, Michigan, United States of America; 10 Department of Biomedical Engineering, New Jersey Institute of Technology, Newark, New Jersey, United States of America; 11 Human Performance and Engineering Laboratory, Kessler Foundation Research Center, West Orange, New Jersey, United States of America; 12 Laboratory of Neuro-Imaging, University of Southern California, Los Angeles, California United States of America; Université catholique de Louvain, Belgium

## Abstract

Regional cortical thickness alterations have been reported in many chronic inflammatory and painful conditions, including inflammatory bowel diseases (IBD) and irritable bowel syndrome (IBS), even though the mechanisms underlying such neuroplastic changes remain poorly understood. In order to better understand the mechanisms contributing to grey matter changes, the current study sought to identify the differences in regional alterations in cortical thickness between healthy controls and two chronic visceral pain syndromes, with and without chronic gut inflammation. 41 healthy controls, 11 IBS subjects with diarrhea, and 16 subjects with ulcerative colitis (UC) underwent high-resolution T1-weighted magnetization-prepared rapid acquisition gradient echo scans. Structural image preprocessing and cortical thickness analysis within the region of interests were performed by using the Laboratory of Neuroimaging Pipeline. Group differences were determined using the general linear model and linear contrast analysis. The two disease groups differed significantly in several cortical regions. UC subjects showed greater cortical thickness in anterior cingulate cortical subregions, and in primary somatosensory cortex compared with both IBS and healthy subjects. Compared with healthy subjects, UC subjects showed lower cortical thickness in orbitofrontal cortex and in mid and posterior insula, while IBS subjects showed lower cortical thickness in the anterior insula. Large effects of correlations between symptom duration and thickness in the orbitofrontal cortex and postcentral gyrus were only observed in UC subjects. The findings suggest that the mechanisms underlying the observed gray matter changes in UC subjects represent a consequence of peripheral inflammation, while in IBS subjects central mechanisms may play a primary role.

## Introduction

Inflammatory bowel diseases (IBD) such as ulcerative colitis (UC) are characterized by chronically recurring symptoms of abdominal pain associated with flares of mucosal inflammation. In contrast, in irritable bowel syndrome (IBS), chronically recurring symptoms of abdominal pain and discomfort occur in the absence of mucosal inflammation or other identifiable nociceptive triggers (therefore referred to as “functional” pain syndromes), and symptom flares are often triggered by psychosocial stressors. It is generally assumed that abdominal pain in UC results initially from inflammation induced peripheral and central sensitization of visceral afferent pathways [1], while symptoms in IBS may reflect primarily an alteration in central pain modulation, including alterations in endogenous descending pain modulation mechanisms [2]. On the other hand, several pieces of evidence support the concept that IBD patients effectively engage endogenous pain inhibition systems [3], including greater engagement of a cortico limbic-pontine pain modulation network compared to IBS subjects [4]. These differences in the engagement of endogenous pain modulation systems may explain the clinical observation that in uncomplicated UC, abdominal pain is not a prominent symptom even during flares.

Several studies have applied multimodal brain imaging to investigate the presence of grey matter changes in patients with various chronic pain conditions without known nociceptive drive [5]–[9], with presumed nociceptive drive [10]–[12], and with known inflammatory drive [13]–[18]. Reported abnormalities in these studies suggest some similarities in findings (e.g. gray matter reduction in insula [INS] and anterior cingulate cortex [ACC] subregions), and increases in CT in somatosensory regions regardless of pain syndrome, and no clear differences have emerged between the different pain categories, in particular between chronic visceral pain of “functional” and of inflammatory origin. The use of different analysis techniques by different groups, makes interpretive comparisons between studies and between different patient populations more difficult.

In the current study, we used the Laboratory of Neuroimaging (LONI) Pipeline [19], [20] for image preprocessing, volumetric analysis and cortical thickness (CT) analysis. We focused on differences of local morphologic brain alterations between UC and healthy control subjects (HCs), and compared them to findings in IBS subjects. Specifically, we aimed to test the following hypotheses: 1) Both IBS and UC patients differ from HCs in terms of regional CT changes. 2) UC patients show CT changes in brain regions involved in somatosensory and viscerosensory processing and modulation. 3) IBS patients show CT changes in brain regions involved in the integration of affective, cognitive and interoceptive signals. 4) In UC patients, there are correlations between CT changes and duration of gut inflammation, reflecting the chronic influence of peripheral inflammation on the brain.

## Materials and Methods

### Subjects

A total 68 right-handed male and female subjects were recruited through the UCLA Digestive Disease Clinic and advertisements including HCs (n = 41; mean age = 28.2 years old, range = 19–48 years; 16 males), IBS with diarrhea (n = 11; mean age = 31.6 years old, range = 21–47 years; 2 males), and UC subjects (n = 16; mean age = 28. 6 years old, range 18–48 years; 10 males). 15 HCs and 5 IBS subjects of the respective samples have been included in a previously published gray matter volume analysis [9]. Exclusion criteria for all subjects comprised pregnancy, postpartum or nursing females, current substance abuse or dependence, abdominal surgery, any past or present neurological illness or trauma, claustrophobia or learning disability, and current psychiatric diagnosis. A diagnosis of IBS was made by a gastroenterologist or nurse practitioner with expertise in functional GI disorders based on the ROME II or ROME III symptom criteria during a clinical assessment [21], [22]. The diagnostic criteria include recurrent abdominal pain or discomfort associated with two or more of the following: 1) pain/discomfort is relieved/improved by defecation 2) the onset of pain/discomfort is related to a change in frequency of stool 3) the onset of pain/discomfort is related to a change in the form (appearance) of stool. In order to match the predominant bowel habit of UC patients, only IBS patients with diarrhea were used in this study. In addition, IBS subjects with current regular use of analgesic drugs (including narcotics, opioids and alpha2-delta ligands) were excluded. UC patients were diagnosed by a gastroenterologist, which was supported by biopsies obtained by endoscopy. During screening, all subjects completed the modified Mayo UCDAI (disease activity index for Stool frequency, rectal bleeding and physician rating of disease activity) to assess degree of current disease. A score of 0–1 is considered Remission, score of 2–4 mild disease, and >4 is considered active disease [23]. Exclusion criteria specific to the UC population were corticosteroid use within last 6 months, or use of any psychotropic medications. All procedures were approved by the UCLA Medical Institutional Review Board, and all subjects provided written informed consent. Questionnaires were completed before scanning to determine symptom type, severity, duration of symptoms, and abdominal sensation (UCLA Bowel Symptom Questionnaire, BSQ) [24], comorbid affective and mood disorders (Hospital Anxiety Depression Scale, HAD) [25], and IBS-related fears and anxiety (Visceral Sensitivity Index, VSI) [26], [27]. Details are shown in Table 1.

**Table 1:** Clinical and behavioral characteristics.

	HCs			IBS subjects			UC subjects			F	Sig.
	N	Mean	SD	N	Mean	SD	N	Mean	SD		
Sex (Male/Female)	16/25			2/9			10/6				
Age	41	28.17	8.43	11	31.55	9.49	16	28.56	8.95	.66	.52
Anxiety symptoms^1^	41	2.8	2.33	11	6.91	3.67	16	7.31	3.55	18.29	<.01
Depression symptoms^1^	41	.85	1.22	11	2.64	2.73	16	3.44	3.54	7.77	<.01
Visceral Sensitivity Index ^2^	40	2.83	5.17	11	37.45	12.94	15	26.13	14.09	61.68	<.01
Overall Bowel Symptoms^3^				11	11.91	2.74	13	4.69	2.46	54.12	<.01
Abdominal Pain^4^				11	10.55	4.3	14	4.14	3.61	18.01	<.01
Abdominal Discomfort^5^				11	11.91	4.21	16	4.06	3.86	49.63	<.01
Duration of symptoms^6^				10	8.5	6.13	16	9.81	9.35	1.67	.69

F =  main effect of group from ANOVA and t-tests for four and two group comparisons, respectively.

1. HAD: Hospital Anxiety and Depression [25];

2. VSI: Visceral Sensitivity Index [26], [27];

BSQ: Bowel Symptom Questionnaire [24].

3. BSQ Overall Symptoms in the Past week (0–20).

4. BSQ Abdominal Pain in the Past week (0–20).

5. BSQ Discomfort in the Past week (0–20).

6. BSQ Duration in years, derived from onset of symptom.

Statistically significant p<0.05.

### Structural MRI Acquisition

All high-resolution T1-weighted brain images were collected at the UCLA Brain Mapping Center using a Siemens 3 Tesla Trio with magnetization prepared rapid gradient echo scanning parameters (TR = 2200 ms, TE = 3.26 ms, flip angle = 9°, duration = 9:03 mins, FOV = 256).

### Data analysis

We employed the LONI pipeline for image preprocessing, cortical surface modeling and gray matter thickness analysis [19], [20]. Following a de-identification step, the structural neuroimaging data were converted from Digital Imaging and Communications in Medicine to ANALYZE 7.5 format, skull-stripped using the LONI Skull-Stripping Meta Algorithm pipeline workflow [28] and cortical surface models were generated using FreeSurfer 4.0 [29] (http://surfer.nmr.mgh.harvard.edu/fswiki and http://ucla.in/xSQPqT). Cortical grey matter thickness was computed at each point of the surface using the distance from the pial surface to the nearest point on the white matter surface. For numerical implementation, we first built the signed distance function [30], [31] of the white matter surface in 3D space and then computed the CT as the value on the signed distance function at those locations. The cortical surfaces and the corresponding CT maps were registered to the International Consortium for Brain Mapping (ICBM) brain surface [32] and then vertex-wise correspondences were established between all cortical surface models using a Conformal Metric Optimization method [33]. An experienced human brain researcher rated each brain surface reconstruction by visually inspecting the surfaces using LONI ShapeViewer (http://www.loni.ucla.edu/Software/ShapeViewer). The quality of surface reconstruction and accuracy of vertex labeling were assessed on the scale of 0 to 1 (0 = completely unacceptable; 1 = perfectly reconstructed and labeled). A threshold of 0.7 was selected as the criterion to reconstruct a subject's surface data to be included in the final analysis.

### Region of interest analysis

We examined the CT change in several manually delineated regions of interest (ROIs) in each hemisphere based on previous studies [11], [14], [34]–[39]. These ROIs included insular subregions (anterior INS [aINS], mid INS [mINS], and posterior INS [pINS]), cingulate subregions (subgenual ACC [sgACC], pregenual ACC [pgACC], anterior midcingulate cortex [aMCC] and posterior MCC [pMCC]), orbitofrontal gyrus (OFG) (lateral OFG and medial OFG), pre- and postcentral gyri [32]. No subregions of pre- and postcentral gyri were drawn. The subregions of INS and cingulum were manually delineated on the 3D ICBM brain atlas [32] by two well-trained researchers with good command of neuroanatomical knowledge (Figure S1). The 3D ROI masks were transformed back onto the ICBM surface space by resampling the atlas map based on masks' Euclidean coordinates [33].

### Statistical analysis

To determine potential protocol differences in ROIs, a general linear model (GLM) was applied to examine differences in total gray matter volumes within HCs as a function of protocol. Group differences in CT within ROIs as a function of group, sex and group*sex were determined using the GLM and weighted linear contrast analysis controlling for total gray matter volume, and age in SPSS v19. The contrasts testing the interaction between group and sex were weighted to eliminate any bias caused by unbalanced representation of sexes. Although this was a hypothesis driven study, we implemented a conservative procedure to adjust for multiple comparisons in order to control for type I error. Specifically, the false-discovery rate (FDR) for the 66 contrasts (11 bilateral ROIs [n = 22] for each of three independent contrasts) was held at 5% [40]–[42]. GLM and linear contrast analysis controlling for sex and age were also applied to examine differences in BSQ between IBS and UC subjects as well as group differences in non-BSQ clinical and behavioral characteristics including VSI and HAD. Significance was determined after controlling FDR at 5% [40], [41].

### Correlation Analysis of Clinic Variables

Within group exploratory partial correlation analyses controlling for total gray matter volume, sex, and age were performed to characterize the association between subjects' clinic characteristics (BSQ, VSI, HAD and Mayo UCDAI) and regions showing significant group differences in CT. However, for the partial correlation between significant ROIs and symptom duration, we did not include age as a covariate, as these explanatory variables were significantly correlated, for IBS group (duration, r = .39, p = .036), and for UC group (duration, r = .67, p = .001) [43]. Significance was determined after controlling FDR at 5% [40], [41].

## Results

### Subject Characteristics

Subjects' clinical data are summarized in Table 1. Compared to the HCs, the two disease groups showed significantly higher measures of symptom related anxiety (VSI scores: IBS>HCs, F = 126.28, p<.001; UC>HCs, F = 71.97, p<.001; IBS>UC, F = 10.61, p = .002), anxiety symptoms (HAD scores: IBS>HCs, F = 17.03, p<.001; UC>HCs, F = 28.02, p<.001), and depression symptoms (HAD scores: IBS>HCs, F = 4.88, p = .031; UC>HCs, F = 15.16, p<.001). IBS subjects had significantly higher overall bowel symptoms scores, more abdominal discomfort and pain than UC subjects. Using the Mayo UCDAI [23], 5 of the UC subjects were in remission, 7 had mild disease and 4 had active disease (mild flare). There were no differences in terms of age of subjects and duration of GI symptoms.

### Regional Cortical Thickness Changes Using ROI Analysis

**Figure 1:**
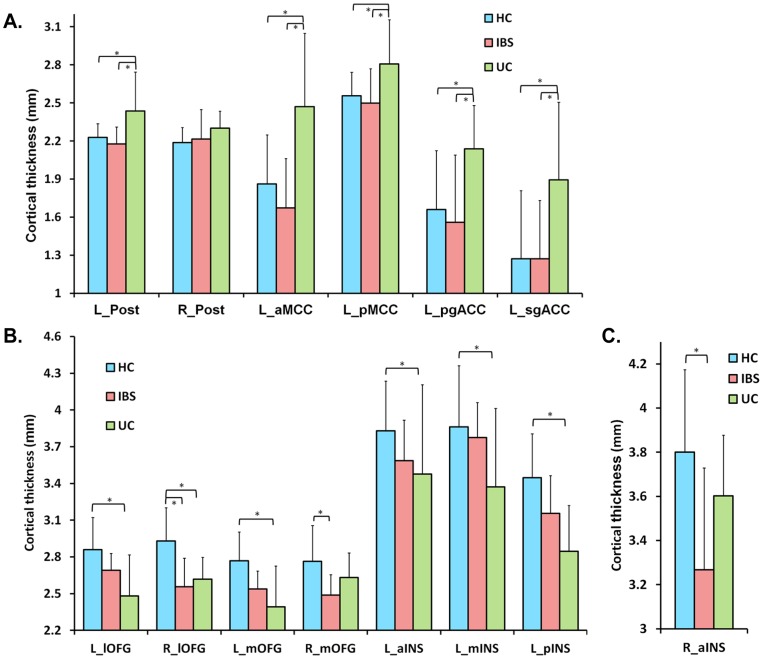
Mean cortical thickness of the ROIs showing significant group differences. (A) UC subjects showed the greatest CT in the regions of somatosensory and cingulate cortex. (B) In the subregions of OFG and INS, UCs had lower CT compared with HCs. (C) IBS subjects had lower CT in right aINS compared to HCs. Error bars reflect standard deviation. Asterisk indicates significant differences between groups (q<0.05) after controlling for sex, age, total gray matter volume and FDR correction.

Regional CT differences were observed between UC, IBS and HCs. Mean CT values and statistical significance after FDR correction are shown in Table 2 and Table S1. As depicted in Figure 1A, compared to both IBS and HCs, UC subjects showed greater CT in left cingulate cortical subregions (aMCC, pMCC, pgACC and sgACC), and in left post central gyrus (statistically significant differences following FDR correction for multiple comparisons are shown in Figure 1A and marked with asterisks). As shown in Figure 1B, compared to both IBS and HCs, UC subjects had reduced CT in prefrontal regions (left medial and lateral OFG) and in left INS subregions (most significant in pINS). Following FDR correction for multiple comparisons, the results between HCs and UC remained significant even though some differences (OFG and INS) between IBS and UC groups were no longer significant (significant differences are shown in Figure 1B and marked with asterisks). All results remained significant after controlling for depression. However, after controlling for anxiety, the observed differences between HCs and UC subjects in left pMCC, aINS and mINS were no longer significant after FDR correction (Table 2). As shown in Figure 1C, compared to HCs, IBS subjects showed significantly reduced CT in right aINS after FDR correction, a difference that was not affected by controlling for anxiety and depression.

**Table 2:** Significant cortical thickness differences in the ROIs between HCs, IBS and UC subjects with and without controlling for anxiety and depression scores.

ROI		Difference	F	P value	q value	F(A)	q(A)	F(D)	q(D)
lOFG	L	HC>UC	23.736	<.00001	.00023	15.898	.00411	23.592	.00020
lOFG	R	HC>UC	15.778	.00019	.00160	9.971	.01654	14.000	.00275
		HC>IBS	17.591	.00009	.00086	12.647	.00925	17.184	.00102
mOFG	L	HC>UC	23.124	.00001	.00023	13.904	.00715	26.905	.00009
mOFG	R	HC>IBS	9.052	.00383	.01686	8.744	.02156	9.802	.01304
Post	L	UC>HC	9.895	.00258	.01216	9.397	.01966	17.446	.00102
		UC>IBS	11.701	.00113	.00678	11.859	.01001	15.595	.00155
aINS	L	HC>UC	6.701	.01207	.04193	2.234	.26245	7.698	.02568
aINS	R	HC>IBS	18.098	.00007	.00082	12.384	.00925	16.893	.00102
mINS	L	HC>UC	10.304	.00213	.01173	3.859	.14306	12.453	.00489
pINS	L	HC>UC	30.701	<.00001	.00005	19.658	.00150	29.416	.00008
aMCC	L	UC>HC	18.231	.00007	.00008	11.277	.01137	18.651	.00080
		UC>IBS	19.862	.00004	.00006	19.387	.00150	21.006	.00040
pMCC	L	UC>HC	8.870	.00042	.01724	2.278	.26245	11.277	.00758
		UC>IBS	7.831	.00690	.02677	7.666	.03185	9.082	.01568
pgACC	L	UC>HC	11.713	.00112	.00678	8.839	.02156	8.901	.01607
		UC>IBS	10.127	.00232	.01175	10.014	.01654	9.690	.01304
sgACC	L	UC>HC	11.754	.00110	.00678	8.692	.02156	9.611	.01304
		UC>IBS	7.704	.00734	.02690	7.609	.03185	7.559	.02611

R: right; L: left; lOFG: lateral orbitofrontal gyrus; mOFG: medial orbitofrontal gyrus; Post: postcentral gyrus; aINS: aINSula; mINS: mid insula; pINS: posterior insula; aMCC: anterior mid cingulate cortex; pMCC: posterior mid cingulate cortex; pgACC: pregenual anterior cingulate cortex; sgACC: subgenual anterior cingulate cortex; q value: p value after FDR corrected at 5%, q value <.05 was considered significant; F(A): F score after controlling for anxiety; q(A): corrected p value after controlling for anxiety; F(D): F score after controlling for depression; q(D): corrected p value after controlling for depression.

### Correlation of Cortical Thickness with Behavioral and Clinical Variables

**Figure 2:**
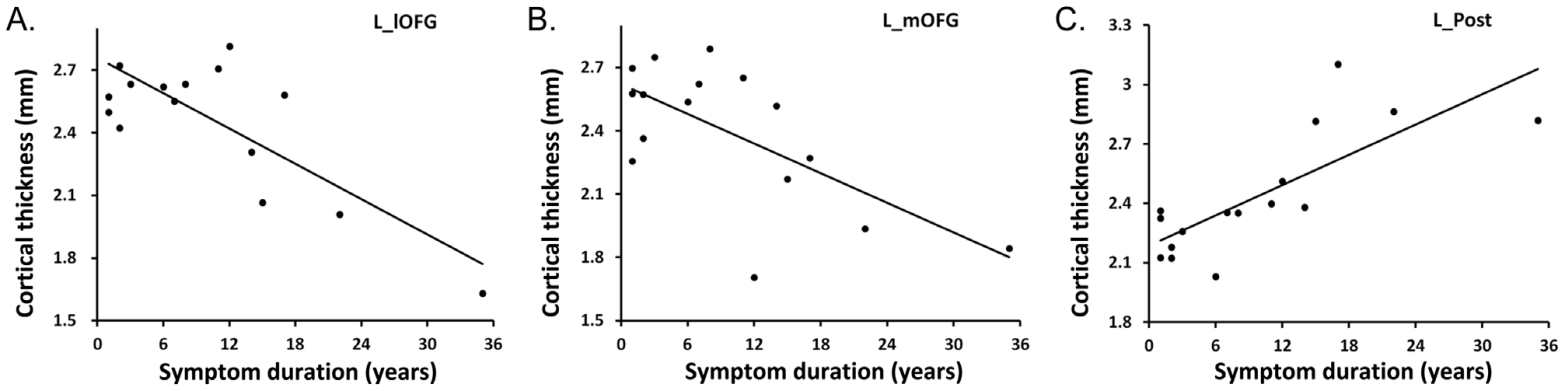
Correlation between cortical thickness and UC symptom duration. (A) Cortical thickness in left lateral orbitofrontal gyrus (L_lOFG) and (B) left medial orbitofrontal gyrus (L_mOFG) were negatively correlated with symptom duration in UC group. (C) Cortical thickness in left postcentral gyrus (L_Post) showed large positive correlation with UC symptom duration.

In the UC group, symptom duration was *negatively* correlated with CT in left lateral OFG (r = −.88, p = .0002, q = .0006, Figure 2A) and left medial OFG (r = −.78, p = .003, q = .003, Figure 2B) and was *positively* correlated with CT in left postcentral gyrus (r = .77, p = .003, q = .003, Figure 2C). Adding anxiety and depression scores as covariates did not alter the results. No significant correlations with other clinical parameters (including the Mayo UCDAI) were observed.

## Discussion

The primary goal of the current study was to assess regional CT differences between subjects with UC, and two comparison groups: a healthy control group and a disease control group without gut inflammation (IBS).The main findings of the study were: 1) Compared to both IBS and HCs, UC subjects showed *greater* CT in left cingulate cortical subregions, and in left primary somatosensory cortex (SI). 2) Compared with HCs, UC subjects showed *lower* CT in left OFG and in primary viscerosensory cortex (pINS). 3) Compared to HCs, IBS subjects showed *lower* CT in the interoceptive association cortex (aINS) in the right hemisphere. 4) There were large significant correlations of CT reductions in left OFG and CT increases in left SI with symptom duration in UC subjects, suggesting a role of chronic inflammation driven afferent input in these changes. The emerging pattern highlights significant differences in CT between patients with chronic gut inflammation, functional GI disorders and HCs, as well as some similarities.

### Greater Regional CT in UC Patients

#### Somatosensory Cortex

In the current study, greater CT in primary somatosensory cortex (SI) was seen in the UC group. SI is part of the central pain processing network and its thickness is positively correlated with individual experimentally induced acute pain sensitivity in healthy subjects [44], [45]. Chronic pain in human patient populations has been shown to be associated with cortical reorganization and changes in SI activity [38], [46], [47]. For example, SI cortical thickening has been reported in patients with migraine [48] and temporomandibular pain [43]. It has been suggested that the critical factor for S1 to undergo structural reorganization may be the presence of constant sensory input to this brain region [38], [43], [47]. Additionally, a voxel based morphometry study showed increased left pre- and postcentral gyri in chronic back pain patients [39]. In the current study, CT of left postcentral gyrus in UC groups showed a large positive correlation with symptom duration, consistent with a possible etiologic role of chronically enhanced viscerosensory input to the brain due to sensitization of visceral afferent pathways by chronic mucosal inflammation. However, the degree of somatosensory cortex changes did not correspond to the subjective pain reports, as UC subjects had greater CT in somatosensory cortex, but reported lower abdominal pain and discomfort compared to patients with IBS. Even though the reason(s) for these apparent discrepancies between CT differences in SI and subjective pain reports are not known, one may speculate that the subjective experience of chronic clinical visceral pain (as opposed to acute experimentally induced pain) is more related to activity and related structural changes in interoceptive association cortex (e.g. the aINS), rather than to primary sensory cortex [4], [49].

#### Midcingulate Cortex

In the current study, compared to HCs and IBS subjects, the UC subjects had greater CT in subregions of the cingulate cortex, e.g. aMCC and pMCC. MCC is involved in emotion processing, skeletomotor regulation, chronic somatic and visceral pain, and along with the aINS (as part of the “salience network”) integrating information to form conceptual pain [50]–[54]. Several studies have reported abnormal MCC activation by acute noxious visceral stimulation in IBS subjects [55]–[58]. Supporting a possible effect of repeated nociceptive stimuli on MCC structure, repeated application of thermal pain stimuli to healthy subjects over a period of 8 days resulted in gray matter increases in both MCC and SI [59]. Together with the observed greater CT in SI, the findings in UC patients are most consistent with the presence of a constant sensory input from the gut, due to sensitization of visceral afferent pathways by chronic mucosal inflammation. This interpretation is also consistent with the fact that in the current study, the IBS group (e.g. without chronically recurring mucosal inflammation) did not show significant CT change compared to HCs. Further support for differential brain mechanisms underlying chronic visceral pain comes from a recent PET ligand study which showed differences in neurokinin-1 receptor (NK-1R) binding potential (e.g. receptor availability) between patients with IBD (including Crohn's disease and UC) and IBS subjects [60]. Compared to HCs, IBD patients had low NK-1R availability in ACC and MCC, while IBS showed this deficit to a lesser extent. Animal studies have shown that the substance P/NK-1R signaling system is involved in cytogenesis, has neurotrophic and neuroprotective functions and inhibits apoptosis [61]–[63]. This implies a differential involvement of such neuroplastic mechanisms in the two visceral pain syndromes. Our findings differ from those reported in two other chronic inflammatory conditions, e.g. Crohn's disease [14] and osteoarthritis [64]. In both of these studies lower gray matter in the MCC was observed compared to HCs. Differences in patient populations and analysis methodology make it difficult to directly compare these studies with the current report [14], [64].

### Reduced Regional CT in IBS and UC Patients

#### INS Subregions

When compared to HCs, both disease groups showed lower CT in the INS, albeit in different subregions. Several studies in patients with chronic pain including IBS [7], [9], [12], [13], [64]–[68] compared to HCs, have found lower gray matter volumes and CT in the INS, even though subregions were often not specified. In the current study, UC compared to HCs had significantly reduced CT in the left pINS (observed differences in mINS were no longer seen after controlling for anxiety). It is likely that chronically enhanced afferent input from the gut due to recurrent mucosal inflammation is primarily associated with CT changes in the pINS, which represents the primary interoceptive cortex [37], [69]. Even though there was no significant correlation between symptom duration or other behavioral measures with CT changes in the pINS, a chronic low back pain study showed that recovery of CT in pINS (and secondary somatosensory cortex) was correlated with reduction of pain intensity after treatment [12], implicating chronic nociceptive and inflammation related signaling as a factor in these CT changes.

In contrast to the UC group, IBS subjects had lower CT in a different subregion of the INS, e.g. the right aINS compared to HCs. The aINS functions as interoceptive association cortex integrates interoceptive input with emotional, salient and cognitive inputs, and provides output to autonomic and pain modulation systems [37], [53], [69]–[71]. The aINS also plays a central role in prediction, error processing, and self awareness of sensations [37], [71]. Even though both patient groups had greater affective scores compared to HCs, IBS subjects reported more abdominal pain and discomfort compared to the UC group. However, there was no significant correlation of the observed changes with affective measures, symptom scores or duration of symptoms in IBS subjects.

#### Orbitofrontal Gyrus

UC subjects compared to HCs showed lower CT in the bilateral OFG, a brain region which plays an important role in interoception, emotion evaluation and regulation, and in cognitive reappraisal [43], [72], [73]. Decreased gray matter in OFG has also been found in other chronic pain disorders with inflammatory/nociceptive drive including hip osteoarthritis [64], low back pain [39] and migraine [74]. A negative correlation between left OFG thickness and symptom duration was observed in UC subjects, suggesting a role of chronic nociceptive input in the observed CT reductions. In contrast, no such correlation between IBS symptom duration and OFG structure were observed.

### Limitations

Limitations of the study include the small sample size of the IBS and UC population, the group differences in level of anxiety and depression symptoms, and the heterogeneity of the groups in terms of sex. However, controlling for anxiety and depression, most of the observed results remained significant. In addition, our GLM and linear contrast were weighted to eliminate any bias caused by umbalanced representation of sexes. Furthermore, the fact that large correlations of some structural changes with disease duration were observed in the UC subjects, and the fact that some of the findings were similar to reports in subjects with other chronic inflammatory conditions [13], [64], makes it unlikely that the findings are confounded by these limitations. Future, longitudinal studies in larger patient populations, including the correlation of plasma and mucosal inflammatory disease markers with structural brain changes both during disease flares and remissions are needed in order to better understand the role of colonic inflammation in remodeling of the brain. In such larger studies, presence of comorbidities in the IBS group, as well as differences in the impact of the IBS and UC on daily life activities and social interactions should be taken into account.

## Conclusions

To our knowledge, this study represents the first comparison of brain structure between UC patients and both HCs and IBS subjects. The findings demonstrate significant differences in CT between UC and HC subjects, and differences between the two disease groups. Based on the correlation of structural changes with symptom duration in IBD, one may speculate that the observed gray matter reorganization of IBD subjects represents a consequence of chronic viscerosensory input to the brain due to sensitization of visceral afferent pathways by recurrent gut inflammation. The mechanisms by which such increased viscerosensory to the brain input can produce both increases and decreases of grey matter in different brain regions remains to be determined.

## Supporting Information

Figure S1 Manually delineated subregions of interest on the 3D International Consortium for Brain Mapping brain atlas. (A) Subregions of cingulate cortex: anterior mid cingulate cortex (aMCC), posterior mid cingulate cortex (pMCC), pregenual anterior cingulate cortex (pgACC) and subgenual anterior cingulate cortex (sgACC). (B) Subregions of insula: aINSula (aINS), mid insula (mINS) and posterior insula (pINS).
(TIF)

Table S1 Mean cortical thickness.
(DOCX)
