# Correction: Regional Neuroplastic Brain Changes in Patients with Chronic Inflammatory and Non-Inflammatory Visceral Pain

**DOI:** 10.1371/journal.pone.0091490

**Published:** 2014-02-28

**Authors:** The PLOS ONE Staff

There were multiple errors in multiple sentences of the “Data Analysis” section of the Materials and Methods. The “Data Analysis” section should read: We employed the LONI pipeline for image preprocessing, cortical surface modeling and gray matter thickness analysis [19], [20]. Following a de-identification step, the structural neuroimaging data were converted from Digital Imaging and Communications in Medicine to ANALYZE 7.5 format, skull-stripped using the LONI Skull-Stripping Meta Algorithm pipeline workflow [28] and cortical surface models were generated using FreeSurfer 4.0 [29] (http://surfer.nmr.mgh.harvard.edu/fswiki and http://pipeline.loni.usc.edu/fsl-feat-4-and-freesurfer/). Cortical grey matter thickness was computed at each point of the surface using the distance from the pial surface to the nearest point on the white matter surface. For numerical implementation, we first built the signed distance function [30], [31] of the white matter surface in 3D space and then computed the CT as the value on the signed distance function at those locations. The cortical surfaces and the corresponding CT maps were registered to the International Consortium for Brain Mapping (ICBM) brain surface [32] and then vertex-wise correspondences were established between all cortical surface models using a Conformal Metric Optimization method [33]. An experienced human brain researcher rated each brain surface reconstruction by visually inspecting the surfaces using LONI ShapeViewer (http://www.loni.usc.edu/Software/ShapeViewer). The quality of surface reconstruction and accuracy of vertex labeling were assessed on the scale of 0 to 1 (0  =  completely unacceptable; 1  =  perfectly reconstructed and labeled). A threshold of 0.7 was selected as the criterion to reconstruct a subject's surface data to be included in the final analysis.
