# Supplementary figures and images for: Regional Neuroplastic Brain Changes in Patients with Chronic Inflammatory and Non-Inflammatory Visceral Pain
Source: PLoS One. 2014 Jan 8;9(1):e84564. doi: 10.1371/journal.pone.0084564 (PMC3885578; doi:10.1371/journal.pone.0084564)

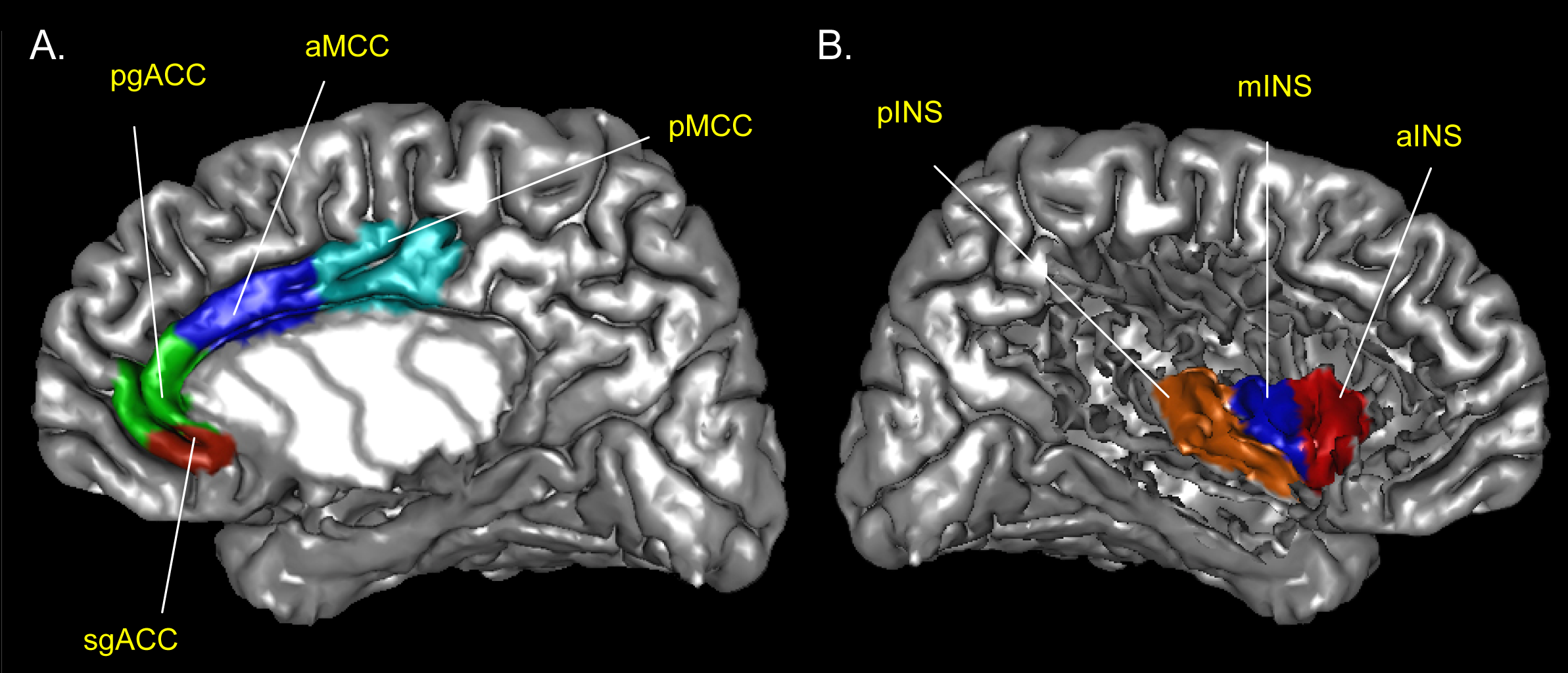

Supplement: Figure S1 — Manually delineated subregions of interest on the 3D International Consortium for Brain Mapping brain atlas. (A) Subregions of cingulate cortex: anterior mid cingulate cortex (aMCC), posterior mid cingulate cortex (pMCC), pregenual anterior cingulate cortex (pgACC) and subgenual anterior cingulate cortex (sgACC). (B) Subregions of insula: aINSula (aINS), mid insula (mINS) and posterior insula (pINS). (TIF) [file pone.0084564.s001.tif]
