# Supplementary material for: Regional Neuroplastic Brain Changes in Patients with Chronic Inflammatory and Non-Inflammatory Visceral Pain
Source: PLoS One. 2014 Jan 8;9(1):e84564. doi: 10.1371/journal.pone.0084564 (PMC3885578; doi:10.1371/journal.pone.0084564)
Supplement: Table S1 — Mean cortical thickness. (DOCX) [file pone.0084564.s002.docx]

**Supplementary Table 1.** Mean cortical thickness

| Group | IOFG | lOFG | mOFG | mOFG | Post | Post | aINS | aINS | mINS | pINS | aMCC | pMCC | pgACC | sgACC |
| --- | --- | --- | --- | --- | --- | --- | --- | --- | --- | --- | --- | --- | --- | --- |
|  | L | R | L | R | L | R | L | R | L | L | L | L | L | L |
| HC | 2.86±.27 | 2.93±.27 | 2.77 ±.24 | 2.76±.29 | 2.23±.11 | 2.19±.12 | 3.83±.41 | 3.8±.37 | 3.86 ±.5 | 3.45±.36 | 1.86 ±.39 | 2.56 ±.19 | 1.66 ±.46 | 1.27 ±.54 |
| IBS | 2.69±.14 | 2.56±.23 | 2.54±.15 | 2.49±.17 | 2.18±.13 | 2.21±.23 | 3.59±.33 | 3.27 ±.46 | 3.78 ±.28 | 3.16±.31 | 1.67 ±.39 | 2.5 ±.27 | 1.56 ±.53 | 1.27 ±.46 |
| UC | 2.48±.34 | 2.62±.18 | 2.39 ±.33 | 2.63±.2 | 2.44±.31 | 2.3 ±.14 | 3.47±.73 | 3.6 ±.27 | 3.37 ±.64 | 2.85±.38 | 2.47 ±.58 | 2.81 ±.35 | 2.14 ±.34 | 1.89 ±.61 |

Mean cortical thickness (mm) ± SD. R: right; L: left; lOFG: lateral orbitofrontal gyrus; mOFG: medial orbitofrontal gyrus; Post: postcentral gyrus; aINS: anterior insula; mINS: mid insula; pINS: posterior insula; aMCC: anterior mid cingulate cortex; pMCC: posterior mid cingulate cortex; pgACC: pregenual anterior cingulate cortex; sgACC: subgenual anterior cingulate cortex
